# An Unusual Case of Chronic Delimited Rhinosinusal Mucormycosis With Secondary Cutaneous Involvement in a Patient With Ataxia-Telangiectasia Syndrome

**DOI:** 10.7759/cureus.88142

**Published:** 2025-07-17

**Authors:** Fadia Y Abdala Mendoza, Diego E Gómez López, Monica Ceballos-Pérez, Circe Ancona Castro, Jorge A García Campos, Rogelio de J. Treviño-Rangel, Hiram Villanueva-Lozano

**Affiliations:** 1 Pediatrics, Instituto de Seguridad y Servicios Sociales de los Trabajadores del Estado (ISSSTE) Regional Hospital, Monterrey, MEX; 2 Dermatology, Instituto de Seguridad y Servicios Sociales de los Trabajadores del Estado (ISSSTE) Regional Hospital, Monterrey, MEX; 3 Infectious Disease, Pediatrics, Instituto de Seguridad y Servicios Sociales de los Trabajadores del Estado (ISSSTE) Regional Hospital, Monterrey, MEX; 4 Microbiology, Faculty of Medicine, Universidad Autónoma de Nuevo León, Monterrey, MEX; 5 Infectious Disease, Internal Medicine, Instituto de Seguridad y Servicios Sociales de los Trabajadores del Estado (ISSSTE) Regional Hospital, Monterrey, MEX

**Keywords:** amphotericin b deoxycholate, ataxia-telangiectasia, cutaneous mucormycosis, “mucormycosis”, rhizopus spp

## Abstract

Mucormycosis is an immunosuppression-related fungal infection. This pathogen usually manifests as an acute and rapidly progressive, deforming, and lethal disease, but chronic forms of zygomycosis occur rarely. Here we present a case of a 15-year-old female with ataxia-telangiectasia syndrome, reporting a six-month history of recurrent sinusitis and an atypical secondary skin infection characterized by ulcerated nodules and crusted plaques, unresponsive to multiple treatments. This case highlights the changing epidemiology of opportunistic infections, which were usually confined to specific immunosuppressive diseases but are recently presenting a shift towards their associated risk factors and comorbidities.

## Introduction

Mucormycosis is an opportunistic fungal infection characterized by a rapid, fulminant progression caused by fungi of the Mucorales group. Most frequently isolated genera are *Rhizopus spp., Mucor spp*., and *Rhizomucor spp.* [1]. Mucorales are mainly responsible for rhinocerebral or rhino-orbital infections, which occur after the inhalation of fungal spores, followed by pulmonary, gastrointestinal, cutaneous, and disseminated forms, predominantly in immunosuppressed patients (e.g., diabetes, renal failure, neutropenia, or the use of steroids) [1,2]. The acute form has been described as a clinical progression, and invasive infection typically occurs rapidly over days without appropriate treatment, although more protracted courses over weeks to months have been reported. The chronic presentations of rhinocerebral mucormycosis have been described [3]. In the chronic infection, the disease course is indolent and slowly progressive, often occurring over weeks to months. The infection can be acute or chronic, depending on the time of evolution, with the chronic form being rare (5.6% of rhinocerebral mucormycosis cases) [4].

Ataxia-telangiectasia (Louis-Bar syndrome) is a rare condition that clinically presents with progressive cerebellar ataxia, dystonia, oculocutaneous telangiectasias, growth retardation, premature aging, recurrent sinopulmonary infections, an increased risk of cancer (particularly of the lymphoid lineage), and hypersensitivity to ionizing radiation. Other clinical manifestations may include hypogonadism and delayed puberty, and nonketotic and insulin-resistant diabetes mellitus. Cellular and humoral immunodeficiency is responsible for the recurrence of upper and lower respiratory tract infections, the latter of which leads to chronic lung disease. Infectious pneumonia (viral or bacterial) or aspiration pneumonia is the leading cause of death in these patients. Cellular and humoral immunodeficiency is a hallmark of this disease, leading to a higher incidence of upper and lower respiratory tract infections [5,6]. However, classic opportunistic infections associated with cellular deficiencies, such as *Pneumocystis jirovecii* pneumonia or *Cryptococcus spp*. meningitis is rarely reported in these patients [7].

We present an unusual case of a patient with ataxia-telangiectasia who developed a chronic rhinosinusoidal lesion with deep and cutaneous involvement caused by *Rhizopus arrhizus.*

## Case presentation

A 15-year-old female with a history of ataxia-telangiectasia syndrome, diagnosed at four years of age, was found to have a confirmed ATM gene mutation with two pathogenic variants (c.2839-3_2839delinsGATACTA (splice acceptor) and c.8977C>T (p.Arg2993)), both in heterozygosis. She also had severe immunodeficiency with IgA and IgE deficiency and severe grade III malnutrition according to WHO criteria.

Six months prior to her current diagnosis, she experienced multiple recurrent episodes of sinusitis, predominantly affecting the left maxillary sinus as confirmed by computed tomography and direct visualization as evaluated by an otorhinolaryngologist. Rhinoscopy revealed a rapidly evolving septal perforation, accompanied by febrile peaks of up to 38°C. Despite the initiation of antimicrobial agents (Table 1) and antifungal regimens (Table 2), no significant clinical response was observed.

**Table 1:** Antimicrobial treatment. PO: oral route; IV: intravenous route; mg: milligram; kg: kilogram

Antimicrobial treatment	Posology dosage	Interval and duration
Ceftriaxone	50 mg/kg/day	IV every 12 hours for 4 days
Ceftazidime	150 mg/kg/day	IV every 8 hours for 6 days
Amikacin	15 mg/kg/day	IV every 12 hours for 7 days
Rifampicin	20 mg/kg/day	IV every 8 hours for 7 days
Vancomycin	43 mg/kg/day	IV every 8 hours for 7 days
Cefepime	160 mg/kg/day	IV every 8 hours for 15 days
Meropenem	75 mg/kg/day	IV every 8 hours for 8 days
Amikacin	15 mg/kg/day	IV every 12 hours for 7 days

**Table 2:** Antifungal treatment. PO: oral route; IV: intravenous route; mg: milligram; kg: kilogram; m^2^: square metre

Antifungal treatment	Posology dosage	Interval and duration
Fluconazole	5.5 mg/kg/day	IV every 24 hours for 7 days
Anmphotericin	1 mg/m^2^/day	IV every 24 hours for 10 days
Fluconazole	8 mg/kg/day	IV every 24 hours for 5 days
Fluconazole	5 mg/kg/day	PO every 24 hours for 14 days

**Figure 1:**
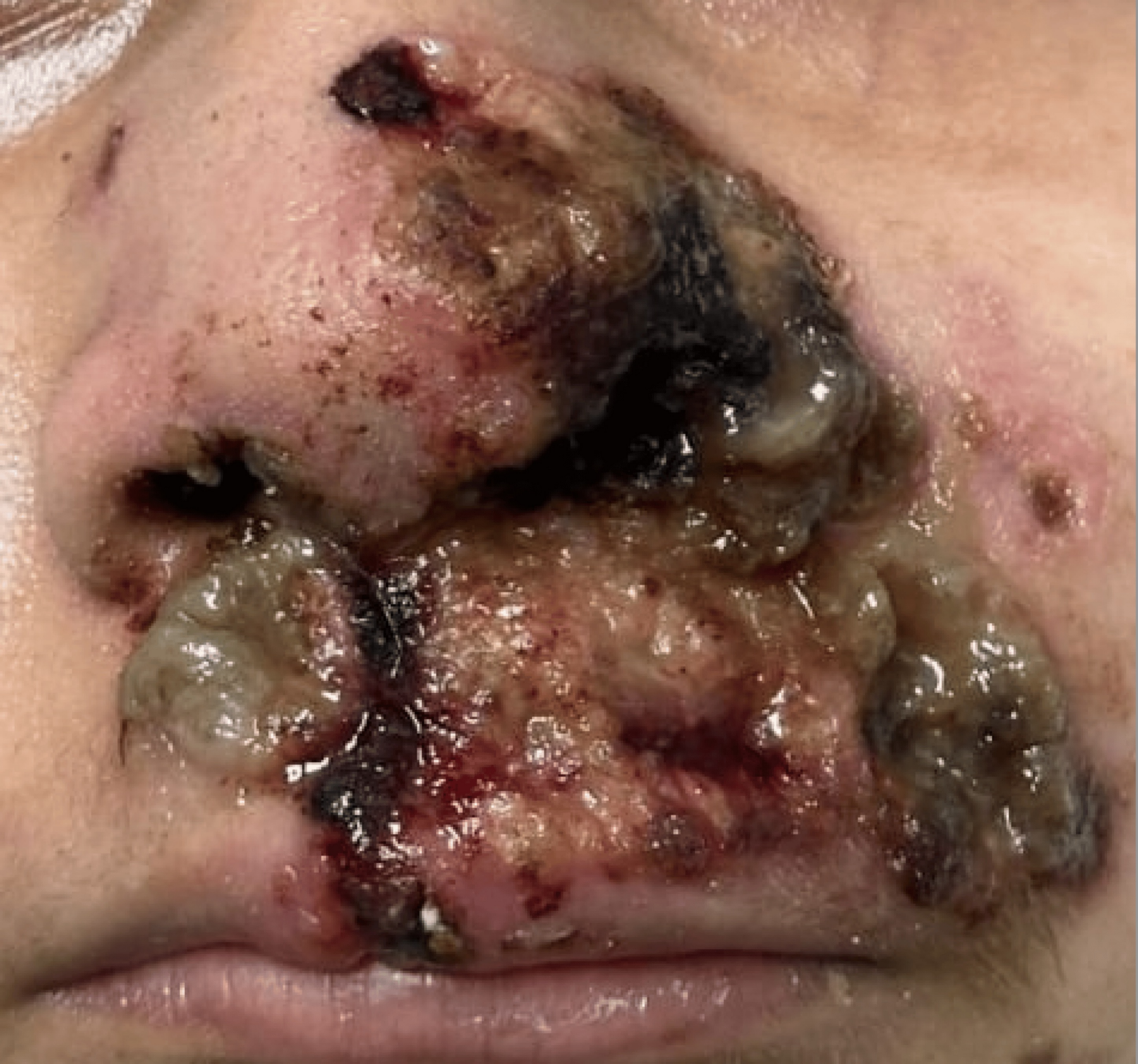
Skin lesions Dermatosis located on the head, affecting the face in the region of the nose, on the left nasal dorsum, extending to the midline, lateral wall, and left nostril orifice, as well as the bilateral alar rim, nasolabial fold, cheek, and medial upper lip. It consists of five ulcerated, necrotic nodules of variable size, ranging from 0.5 to 5 cm in diameter, with well-defined, irregular borders, all settled on an erythematous background. The increased erythema in the background is possibly due to ataxia-telangiectasia. At the periphery of all ulcers, there is granulation tissue; in the background of some ulcers, the presence of fibrin is especially noteworthy at the alar border and nasolabial fold, with a loss of anatomy of the left nasal ala.

Four months later, she developed localized dermatosis on the face, particularly in the nasal region, characterized by nodules that ulcerated within two weeks (Figure 1).

**Figure 2:**
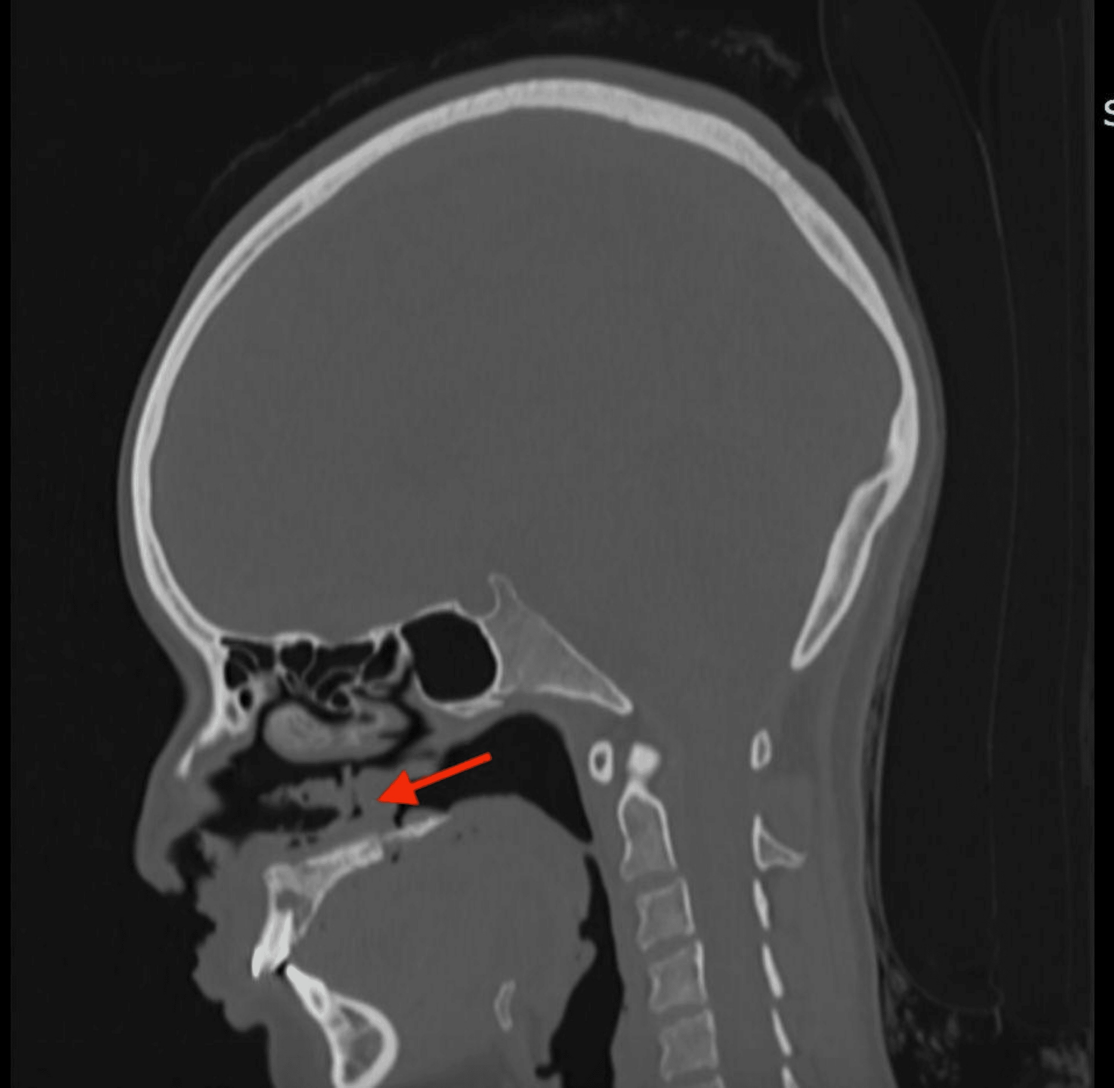
CT scan Sagittal computed tomography: Disruption of soft tissues in the left upper labial region, nasolabial fold, and nasal ala, predominantly on the left side, with perforation of the cartilaginous portion of the nasal septum. There was also the presence of material with soft-tissue density and a gaseous appearance in the nasal and oral vestibule, secondary to perforation of the soft palate (red arrow). Bone tissue shows areas of sclerosis and lytic lesions in the alveolar and palatine processes of the maxillary bone, affecting the posterior surface of the upper incisors, the anterior surface of both maxillary bones, and the horizontal plate of the palatine bone.

She also exhibited soft palate perforation. Through biopsy with special stains, acid-fast bacilli infections were ruled out. Computed tomography findings included disruption of soft tissues at the left upper labial region, nasolabial fold, and nasal ala, predominantly on the left side, with perforation of the cartilaginous portion of the nasal septum. There was also the presence of material with soft tissue density and a gaseous appearance in the nasal and oral vestibule, secondary to perforation of the soft palate. Bone tissue showed areas of sclerosis and lytic lesions in the alveolar and palatine processes of the maxillary bone, affecting the posterior surface of the upper incisors, the anterior surface of the bilateral maxillary bone, and the horizontal lamina of the palatine bone (Figure 2).

Serum protein electrophoresis was performed as part of the diagnostic evaluation to rule out acute or chronic inflammatory conditions, hepatic dysfunction, and paraneoplastic syndromes. Serum protein electrophoresis revealed an alpha-2 fraction of 14.4% (reference range: 7.5-12.6%) and a gamma fraction of 29.2% (reference range: 8.0-15.8%), both of which were positive. 

**Figure 3:**
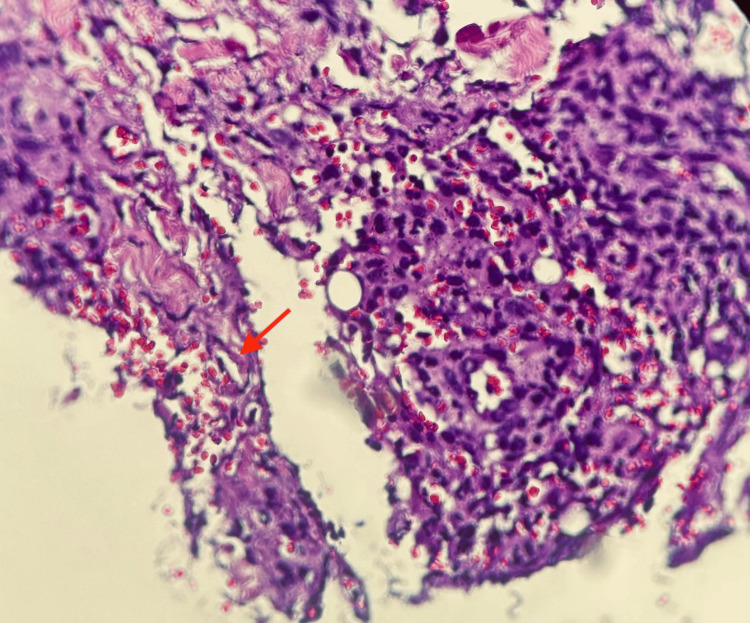
Skin biopsy Gross description: Spindle-shaped skin biopsy measuring 0.7 × 0.4 cm, processed and stained with hematoxylin and eosin. Microscopic description: At the subcutaneous level, there is a mixed inflammatory infiltrate with areas of necrosis, accompanied by numerous macrophages and extravasated erythrocytes. Broad, non-septate hyphae (red arrow) with right-angled (90º) branching are also observed.

Due to its chronic, persistent progression, a new biopsy of the lesion was performed and sent for culture and periodic acid-Schiff (PAS) staining. In culture, a rapidly growing filamentous fungus in Sabouraud-Dextrose and Papa Dextrose agar was visualized, and the presence of broad, irregularly branched, and poorly septate hyphae was reported in histopathology (Figure 3).

**Figure 4:**
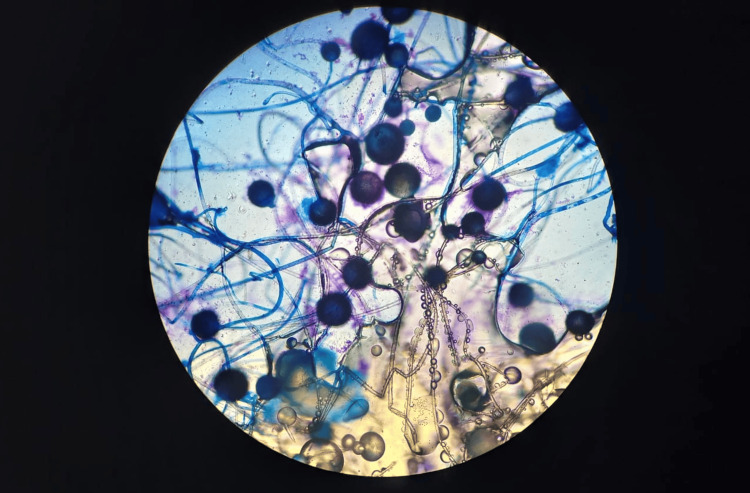
Direct examination Direct examination with lactophenol cotton blue stain revealed numerous fungal structures, with the presence of broad, poorly septate, and branched hyphae, compatible with *Rhizopus spp.*

The fungus was morphologically identified as *Rhizopus spp*. Subsequent sequencing of the internal transcribed spacer (ITS) non-coding region confirmed it as *Rhizopus arrhizus* (Genbank accession number PV558343) (Figure 4).

Antifungal susceptibility testing using the broth microdilution method (Clinical and Laboratory Standards Institute M38-Ed3) [8] was performed, and the following results were presented: itraconazole 8.0 mcg/mL, posaconazole 0.25 mcg/mL, isavuconazole 2.0 mcg/mL, and amphotericin B 0.25 mcg/mL.

**Figure 5:**
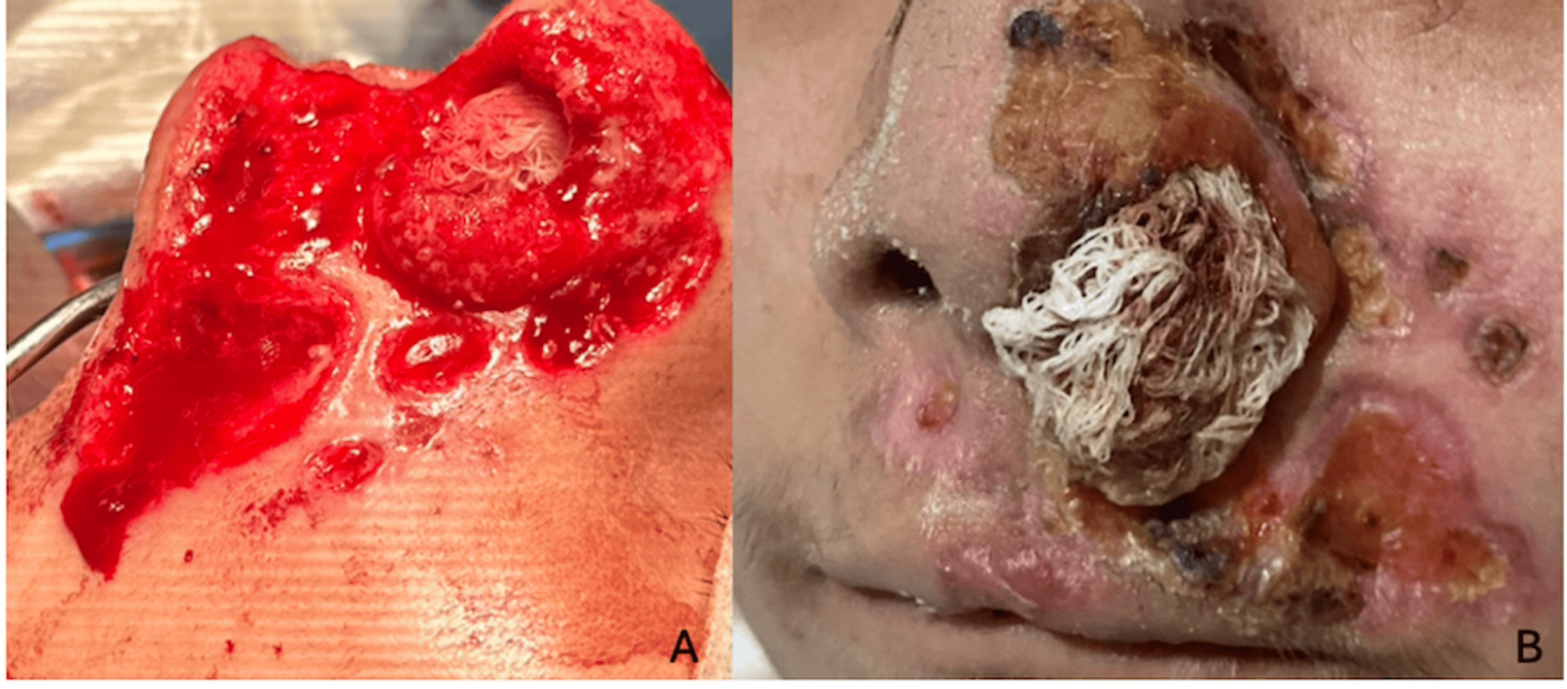
Treatment A: debridement after surgery of the necrotic ulcers; B: evolution one month after debridement, topical treatment with Triticum vulgare and pirfenidone, led to an improvement of the dermatosis, with involution and decrease in the diameter of some of the ulcers.

Due to the limitations in Mexican public hospitals, treatment with amphotericin B deoxycholate (1 mg/kg/day) for 14 days was initiated, and 15 days after the initial dose of amphotericin B, itraconazole oral treatment was started and continued, along with broad-spectrum antibiotics to mitigate the risk of bacterial superinfection. Surgical debridement of necrotic tissue was also performed as a partial debridement (Figure 5A). This was delayed because of the difficulty in obtaining a positive culture/biopsy, as well as diagnostic uncertainty associated with the low reported incidence of this type of localized chronic presentation in this group of patients. The treatment was followed by wound healing with Triticum vulgare and pirfenidone (Figure 5B). The infection was contained, with no systemic involvement confirmed by multiple negative control cultures and bronchoalveolar lavage tests.

Unfortunately, the patient eventually succumbed to complications of her underlying disease.

## Discussion

Acute rhinomucormycosis typically presents as an erythematous and edematous plaque with central ulceration, progressing rapidly to involve subcutaneous tissue, muscle, and bone [1]. However, atypical and chronic forms can also occur, as seen in this patient, who developed rhinosinusal lesions followed by cutaneous nodules that ulcerated [4,6].

In this case, the initial site of infection was the nasal septum, leading to perforation, cutaneous extension, and eventual perforation of the soft palate, creating a communication between the nasal and oral cavities. No systemic, orbital, cerebellar, or other organ involvement was observed.

Severe IgA deficiency likely contributed to the patient’s slow response to treatment, as IgA plays a crucial role in mucosal immunity, preventing pathogen adherence and penetration [9]. The presence of midfacial nodules initially raised suspicion for natural killer (NK) lymphoma due to the rapid clinical progression, which, alongside acute mucormycosis, was considered in the differential diagnosis. However, the slower evolution and chronic nature of the patient’s condition ruled out these acute pathologies.

Considering the markedly chronic progression of the disease, along with the absence of documented cases of mucormycosis associated with ataxia‑telangiectasia syndrome and the exceptional rarity of chronic mucormycosis in pediatric patients, alternative infectious etiologies were contemplated as contributors to the severity of the clinical presentation (Table 3).

**Table 3:** Records of 100 consecutive patients with ataxia-telangiectasia from the Johns Hopkins Ataxia-Telangiectasia Clinical Center (ATCC) reviewed Adapted from [10]

Opportunistic infections encountered with AT	Percentage
Otitis media	46%
Sinusitis	27%
Bronchitis	19%
Neumonía	15%
Sepsis	5%
Herpes simple	8%
Molluscum contagiosum	5%
Candidal esophagitis	3%
Herpes zoster	2%
Uncomplicated varicella	44%

Differential diagnoses included actinomycosis, as well as infections caused by typical and atypical mycobacteria. Despite multiple histopathological and microbiological evaluations, including biopsies obtained from sites such as septal perforations and cutaneous lesions, all specimens were negative for infectious agents at the time of analysis.

Mucormycosis should be included in the differential diagnosis for patients of this profile, particularly underscoring the need for heightened clinical suspicion in cases where the primary disease process has precipitated significant malnutrition and subsequent immunosuppression. This case stands out as a rarely documented chronic mucormycosis in ataxia-telangiectasia syndrome, with unusual skin involvement. Chronic mucormycosis is possible in immunodeficient states. Skin signs can be sentinel clues. High suspicion is crucial despite slow progression.

Infections in immunocompromised patients often present atypically, potentially delaying diagnosis without a high index of suspicion. Early diagnostic methods, such as potassium hydroxide (KOH) or calcofluor staining, are essential for the timely initiation of treatment, which should include surgical debridement and first‑line antifungals like liposomal amphotericin B [1,11,12].

Unfortunately, our patient experienced additional complications, including nosocomial pneumonia and multiorgan failure, leading to hepatic and renal involvement and eventual fatality.

## Conclusions

This case highlights the diagnostic and therapeutic challenges posed by chronic rhinomucormycosis in immunocompromised pediatric patients, particularly in the context of an underlying primary immunodeficiency. The unusual chronic evolution, absence of systemic dissemination, and progressive local tissue destruction underscore the importance of maintaining a high index of suspicion for mucormycosis, even in atypical clinical scenarios. Despite extensive microbiological and histopathological investigations, diagnostic confirmation may remain elusive, reinforcing the need for early empirical antifungal therapy in suspected cases. In patients with severe immunodeficiencies such as IgA deficiency and complex syndromic backgrounds like ataxia-telangiectasia, infections may present insidiously and progress despite standard interventions. Prompt initiation of both surgical and medical treatment remains critical; however, this case tragically demonstrates that advanced disease and comorbidities, such as nosocomial infections and multiorgan failure, can significantly compromise prognosis. Ultimately, early recognition and multidisciplinary management by the ENT surgeons, infectious disease specialists, pathologists, and pediatric or immunology teams are key aspects of complex case care. It's essential to improving outcomes in similarly complex clinical presentations.
